# Epidemiology and outcomes of Endophthalmitis in chronic dialysis patients: a 13-year experience in a tertiary referral center in Taiwan

**DOI:** 10.1186/s12882-017-0684-5

**Published:** 2017-08-16

**Authors:** George Kuo, Yueh-An Lu, Wei-Chiao Sun, Chao-Yu Chen, Huang-Kai Kao, YuJr Lin, Chia-Hui Lee, Cheng-Chieh Hung, Ya-Chung Tian, Hsiang-Hao Hsu

**Affiliations:** 1 Department of Nephrology, Kidney Research Center, Linkou Chang Gung Memorial Hospital, Chang Gung University, College of Medicine, No.5 Fu-Shin Street, Kwei-shan, Taoyuan, 333 Taiwan; 2 Department of Plastic and Reconstructive Surgery, Linkou Chang Gung Memorial Hospital, Chang Gung University, College of Medicine, Taoyuan, Taiwan; 3 Center for Big Data Analytics and Statistics, Linkou Chang Gung Memorial Hospital, Chang Gung University, College of Medicine, Taoyuan, Taiwan; 4 Department of Pharmaceutical Materials Management, Taoyuan Chang Gung Memorial Hospital, Taoyuan, Taiwan

**Keywords:** Blurred vision, Chronic dialysis, Endophthalmitis, Outcome, Ophthalmalgia

## Abstract

**Background:**

Endophthalmitis is a severe eye infection leading to disabling outcome. Because there were only a few case report illustrating endophthalmitis in chronic dialysis patient, we would like to investigate the epidemiology and clinical features of endophthalmitis in chronic dialysis patient in a tertiary referral center.

**Methods:**

We searched the health information system in the study hospital with ICD9 encoding endophthalmitis during Jan. 2002 to Dec. 2015. A total of 32 episodes of endophthalmitis occurred in chronic dialysis patients. We performed an 1:2 case-control match on propensity score. The demographic features, clinical manifestation, infection focus and visual outcome were recorded.

**Results:**

Of the total of 32 patients, 25 were classified as endogenous endophthalmitis and another seven were exogenous. Most patients presented with ophthalmalgia (*n* = 32, 100%) and periocular swelling (*n* = 31, 96.8%), whereas half of the patients suffered blurred vision (*n* = 16, 50%). *Staphylococcus aureus*, *Klebsiella pneumoniae*, and *Pseudomonas aeruginosa* were the most frequent causative pathogens. Dialysis vascular infection was also a possible unique focus for bacteremia. The visual acuity of the endogenous groups were less likely to improve in the chronic dialysis patients compared with control group.

**Conclusion:**

This is the first and the largest case series focusing on endophthalmitis in chronic dialysis patients. Our study showed different pathogen spectrum, an unique bacterial origin and worse visual outcome in these group of patients. Prompt referral to ophthalmologists when the patients present with suspicious symptoms (blurred vision, ophthalmalgia and periocular swelling) is crucial.

## Background

Endophthalmitis is a serious ocular infection that frequently leads to blindness, and it can be further divided into endogenous and exogenous, based on the presence of ocular trauma or surgery prior to the onset of eye infection [1, 2].

Endogenous endophthalmitis usually presents with blurred vision and ocular pain, and only less than half of patients have systemic infectious signs including fever [3]. Retrospective studies demonstrated significant risk factors of endogenous endophthalmitis including diabetes, intravenous drug user, immunosuppressive therapy, active malignancies, and cardiac disease [3–8]. Exogenous endophthalmitis, especially those occur after cataract surgery, is widely described. The risk factors include diabetes, male gender, old age and lack of prophylactic perioperative antibiotics [9–11].

Despite extensive literatures in the general population, there are few studies and reports featuring this serious infection in chronic dialysis patients, of which the immunity is impaired and bloodstream infections are common [12–14]. We here described the largest series of endophthalmitis among chronic dialysis patients in a tertiary referral medical center in Northern Taiwan.

## Methods

The study was carried on at Linkou Chang-Gung Memorial Hospital (CGMH), a tertiary referral centre with about 3700 beds and an average of 107,00 episodes in-patient service annually. This study protocol was approved by the Institutional Review Board (IRB) of the study hospital (Institutional Review Board of Chang Gung Medical Foundation, approval number: 201600974B0). The need of informed consent was waived because of the lack of different intervention to patient groups and no breach of patient privacy.

With the approval of IRB, we accessed and searched the health information system (HIS) of Linkou Chang-Gung Memorial Hospital (CGMH) during Jan. 2002 to Dec. 2015 by using the diagnosis codes (ICD9: 36,000, 36,001, 36,002, 36,019) from the discharge summary of all admission patients. The records of 1145 consecutive patients with diagnosis codes of endophthalmitis from January 2002 to December 2015 were reviewed retrospectively. Patients with end stage renal disease (ESRD) received chronic dialysis that developed endophthalmitis were included. Patients younger than 18-year-old or those undergone solid organ or hematologic stem cell transplant were excluded. The demographic, clinical and laboratory data were collected. The patients were considered cancer/malignancy-free if no relevant information obtained from the HIS or he/she has survived more than 5 years after cancer treatment. The ongoing treatment modality for those with active cancer were also recorded. After excluding patients with abnormal renal function (serum creatinine >1.0 mg/dL) from the previously mentioned 1145 patients with endophthalmitis, we performed an 1:2 case-control match on propensity score dependent on age, sex and the presence of diabetes mellitus.

The visual acuity was expressed as a Snellen test measured at 6-m distance (6/6 vision). Visual acuity below 6/60 was further divided into four categories with a descending order, i.e., count fingers (CF), hand movement (HM), perception of light (PL) and no perception of light (NPL). The visual outcomes were categorized into four groups: improvement, stationary, deterioration, or enucleation. Improvement of visual acuity was defined as a gain of more than two lines of Snellen visual acuity or a one-step improvement of the “below Snellen” scales (e.g. PL to HM, or CF to 6/60). Deterioration was defined as a loss of more than two lines of Snellen visual acuity or a one-step worsening of the “below Snellen” scales (e.g. 6/60 to CF, PL to NPL, or CF to HM). Patients with endophthalmitis were further classified into endogenous or exogenous group. The exogenous group was defined as those received within 6 weeks after ophthalmologic surgery or eyeball trauma. The rest of patients were classified into endogenous group.

### Statistical analysis

The categorical variables are presented with proportion and are compared by Chi-square test. The continuous variables including age, C-reactive protein and white blood count level are presented with mean and standard deviation, and these variables are tested by independent T-test. Variables such as post-operative duration and the duration between symptom onset to the day evaluated by ophthalmologist are presented as a range and median. All statistical analysis are performed by SPSS version 17.

## Results

We enrolled 1145 hospitalized patients with a discharge diagnosis coding of endophthalmitis from a total of 1,494,342 admission episodes since January 1, 2002 till December 31, 2015 (ICD9 code: 36,000, 36,001, 36,002, and 36,019). A total of 41 episodes of endophthalmitis were found in patients received dialysis procedure during their hospitalization. After thorough chart review, nine episodes were excluded (one was pediatric patient, two patients had acute kidney injury, one suffered endophthalmitis before entering ESRD, one with glaucoma rather than endophthalmitis, and four with erroneous coding from previous episode). Of the 32 chronic dialysis patients entering final analysis, 25 were classified into endogenous whereas the other 7 were exogenous. The clinical information of these patients were summarized in Tables 1 and 2 for endogenous and exogenous endophthalmitis, respectively. After propensity score matching based on age, sex and DM, a total of 57 patients with normal renal function were sorted as control group, with 31 endogenous and 26 exogenous endophthalmitis. The demographic data of the dialysis and control group were summarized in Table 3.

**Table 1:** Clinical summary of 25 chronic dialysis patients with endogenous endophthalmitis

No.	Gender	Age	Eyes involved	Immuno-compromised status	Bacteremia	Vitreous culture	Initial visual acuity	Final visual acuity	Treatment	Concomitant infection focus & signs
1	M	87	Right	cirrhosis, active HCC		Kp	-	Enucleation	Enucleation	
2	M	51	Right			N/A	CF	CF	Parenteral antifungus	
3	M	67	Right	DM	ORSA	N/A	-	-	Parenteral antibiotics	TCC infection
4	M	53	Both	DM	OSSA	N/A	CF	CF	Parenteral antibiotics	
5	F	69	Left	DM	CoNS	CoNs	-	-	IVI,TPPV	AVG infection
6	F	56	Left			negative	PL	PL	Parenteral antibiotics	
7	F	84	Right	DM		PsA	NPL	NPL	IVI	
8	M	66	Right	DM		negative	NPL	NPL	IVI	
9	F	70	Right	DM		ORSA	HM	HM	Parenteral antibiotics	
10	M	59	Left	DM		negative	CF	CF	Parenteral antibiotics	
11	M	60	Left	DM	ORSA, PsA	PsA	N/A	N/A	Parenteral antibiotics	Pneumonia
12	F	75	Left			Kp, CoNS	6/60	6/20	IVI,TPPV	
13	M	61	Left	DM		negative	NPL	NPL	IVI	
14	F	86	Right		Bacillus cerues	negative	NPL	Enucleation	Enucleation	TCC infection
15	F	71	Left	DM		negative	N/A	Enucleation	Enucleation	
16	F	70	Right	DM		Fungal: Acremonium	HM	CF	IVI	
17	F	38	Right	Prednisolone 5 mg/day		N/A	6/20	6/20	Parenteral antibiotics	PAOD with gangrenes
18	F	54	Right	DM	OSSA	negative	PL	PL	IVI	TCC infection
19	M	62	Left	DM		negative	CF	CF	IVI	DM foot infection
20	F	52	Both	DM		Kp	N/A	CF	IVI	UTI
21	M	80	Right	DM	PsA	PsA	N/A	Enucleation	Enucleation	
22	M	66	Left	DM, cirrhosis, active HCC		negative	NPL	NPL	IVI	
23	M	46	Right	DM		negative	NPL	NPL	IVI	
24	F	78	Right	nil		negative	NPL	Enucleation	Enucleation	
25	F	49	Right	DM	ORSA	*Staphylococcus epidermidis* *Pseudomonas fluorescens*	6/21	CF	IVI	

*DM* diabetes mellitus, *HCC* hepatocellular carcinoma, *ORSA* oxacillin-resistant *Staphylococcus aureus*, *OSSA* oxacillin-susceptible *Staphylococcus aureus*, *CoNS* coagulase negative *Staphylococcus*, *PsA* Pseudomonas aeruginosa, *Kp Klebsiella pneumoniae*, *IVI* intra-vitreous injection, *TPPV* trans pars plana vitrectomy, *TCC* tunneled cuffed catheter, *AVG* arterio-venous graft, *PAOD* peripheral arterial occlusive disease, *UTI* urinary tract infection, CF count fingers, *HM* hand movement, *PL *perception of light, *NPL* no perception of light, *N/A* not assessable because of impaired consciousness, − missing data

**Table 2:** Clinical summary of 7 chronic dialysis patients with exogenous endophthalmitis

No.	Gender	Age	Eyes involved	Immunocompromised status	Vitreous culture	Surgery before infection	Initial visual acuity	Final visual acuity	Treatment
1	M	47	Right	DM	Negative	Cataract surgery	-	-	Parenteral antibiotics
2	M	60	Left	DM	ORSA	Cataract surgery	-	-	IVI,TPPV
3	F	67	Right	Nil	NFGNB	Cataract surgery	6/30	6/30	IVI
4	M	56	Right	DM	*Enterococcus faecalis*	Cataract surgery	PL	CF	IVI
5	M	64	Left	DM	*Stenotrophomonas maltopilia*	Cataract surgery	CF	6/15	IVI
6	M	73	Right	DM	negative	Cataract surgery	CF	CF	IVI,TPPV
7	M	66	Left	DM, cirrhosis, active HCC	*Enterococcus faecalis*	TPPV with endo-laser for vitreous hemorrhage	HM	NPL	IVI,TPPV

*DM* diabetes mellitus, *HCC* hepatocellular carcinoma, *ORSA* oxacillin-resistant *Staphylococcus aureus*, *NFGNB* glucose non-fermenting Gram negative bacilli, *IVI* intra-vitreous injection, *TPPV* trans pars plana vitrectomy, *PL* perception of light, *NPL* no perception of light, − missing data

**Table 3:** Demographic data of chronic dialysis patients versus patients with normal renal function

	Dialysis				Control			
	Endogenous (*N* = 25)		Exogenous (*N* = 7)		Endogenous (*N* = 31)		Exogenous (*N* = 26)	
Male	12	48.0%	6	85.7%	16	51.6%	13	50.0%
Age	64	±13	62	± 8	61	± 12	67	± 13
Co-morbidities								
Diabetes mellitus	18	72.0%	6	85.7%	24	77.4%	14	53.8%
Hypertension	20	80.0%^#^	5	71.4%	13	41.9%	10	38.5%
Coronary artery disease	7	28.0%^#^	1	14.3%	1	3.2%	0	0%
Congestive heart failure	6	24.0%^#^	1	14.3%	0	0%	0	0%
Stroke	4	16.0%	2	28.6%*	1	3.2%	0	0%
Hepatitis B	1	4.0%	0	0%	2	6.5%	1	3.8%
Hepatitis C	3	12.0%	2	28.6%*	2	6.5%	0	0%
Cirrhosis	2	8.0%	1	14.3%	4	12.9%	0	0%
Cancer	3	12.0%	1	14.3%	3	9.7%	0	0%
Immunosuppression	1	4.0%	0	0%	0	0%	0	0%
Vascular access	HD = 24^+^, PD = 1	HD = 7						
AVF	15	62.5%	7	100%				
AVG	1	4.1%	0					
TCC	9	37.5%	0					

*AVF* native arterio-venous fistula, *AVG* arterio-venous graft, *TCC* tunneled cuffed catheter, *HD* hemodialysis, *PD* peritoneal dialysis

^+^One hemodialysis patient in the endogenous group had both AVF and TCC

^#^ *p* < 0.05 Endogenous: Dialysis v.s. Control

*p < 0.05 Exogenous: Dialysis v.s. Control

Diabetes mellitus was the most frequent co-morbidity across all groups. Hypertension, coronary artery disease and congestive heart failure were more frequent in the dialysis, endogenous endophthalmitis group compared with control (*p* < 0.05). Only one dialysis patient in the endogenous group takes prednisolone 5 mg per day for underlying systemic lupus erythematosus (SLE). Three dialysis patients in the endogenous and one patient in the exogenous group had active hepatocellular carcinoma (HCC), and all of them received local treatment with repetitive radiofrequency ablation or trans-arterial embolization. One dialysis patient in the endogenous group had both completely treated liver and urinary tract cancer and was in disease free status. Three patients in control group had active cancer (1 with hepatocellular carcinoma, 1 with bladder urothelial carcinoma, and 1 with eyelid basal cell carcinoma).

The clinical manifestations of endophthalmitis are summarized in Table 4. The right eye was numerically more frequently involved in the dialysis group compared with control, but this difference did not reach statistical significance. Periocular swelling and ophthalmalgia were more frequently seen in the dialysis than control group, in both endogenous and exogenous endophthalmitis (*p* < 0.05). The median duration from symptom onset to the visit of ophthalmologist were 2 days in dialysis group and 3 days in control group. For endogenous endophthalmitis, the proportion of fever, shock, respiratory failure, or bacteremia were not statistically different in dialysis and control group. The initial WBC were numerically higher in the endogenous than exogenous endophthalmitis but did not reach statistical significance. The CRP level were significantly higher in the endogenous than exogenous endophthalmitis in the control group (*p* < 0.05) but not in the dialysis group (*p* = 0.065). In dialysis patients with endogenous endophthalmitis, three patients with tunneled cuffed catheter (TCC) and one patient with arterio-venous graft (AVG) had evidences of dialysis vascular access infection at the presentation of endophthalmitis. Eight patients in the endogenous group received only parenteral antibiotic treatment because of various reasons including less severe intraocular involvement, patient refusal, or contraindication such as severe thrombocytopenia.

**Table 4:** Clinical presentations and treatments for chronic dialysis patients versus patients with normal renal function

	Dialysis				Control			
	Endogenous (*N* = 25)		Exogenous (*N* = 7)		Endogenous (*N* = 31)		Exogenous (*N* = 26)	
Clinical & laboratory								
Days from symptom onset to ophthalmologists’ visit	1–30 (Median: 2 days)		1–7 (Median: 2 days)		1–30 (Median: 3 days)		1–30 (Median: 3 days)	
Recent trauma or surgery	0	0%	7	100.0%	0	0%	26	100.0%
Days post eye surgery			3–20 (Median: 4 days)				1–35 (Median: 4 days)	
Blurred vision	14	56%	2	28.5%	26	83.9%	25	96.2%
Ophthalmalgia	25	100%^#^	7	100%*	22	71.0%	20	76.9%
Peri-ocular swelling	25	100%^#^	6	85.7%*	10	32.2%	2	7.6%
Fever	8	32.0%	0	0%	17	54.8%	0	0%
Septic shock	2	8.0%	0	0%	5	16.1%	0	0%
Respiratory failure	2	8.0%	0	0%	1	3.2%	0	0%
Dialysis access infection	4	14.8%	0	0%	0	0%	0	0%
Leukocytosis	11	± 5	10	± 6	10	± 4	11	± 5
Serum CRP level	71	± 75	16	± 10	133	± 105	43	± 56
Microbiology								
Bacteremia	8	32.0%	0	0%	13	41.9%	0	0%
Fungemia	0	0	0	0%	0	0%	0	0%
Positive vitreous culture	13	52.0%	4	57.1%	18	58.1%	19	73.1%
Treatment								
IVI	12	48.0%^+^	7	100%	29	93.5%	25	96.2%
TPPV	2	8.0%	3	42.9%	2	6.5%	10	38.5%
Enucleation	5	20.0%	0	0%	6	19.4%	1	3.8%
Parenteral antibiotic only	8	32.0%	0	0%	0	0%	0	0%

*CRP* C-Reactive protein, *IVI* intra-vitreous injection, *TPPV* trans pars plana vitrectomy

^#^ *p* < 0.05 Endogenous: Dialysis v.s. Control

*p < 0.05 Exogenous: Dialysis v.s. Control

^+^ *p* < 0.05 Endogenous: Dialysis v.s. Control

The outcomes regarding visual acuity were summarized in Table 5. There were missing data of 5 patients in the earliest era (2002–2003). Another two patients in the endogenous group were unable to evaluate visual acuity because of impaired consciousness at presentation. After treatment, visual acuity of most patients in both group remained stationary, while only a small fraction of them achieve some degree of improvement. Most of patients in endogenous group had final visual acuity below 6/60 (76%), while 42.8% of patients in exogenous group had final visual acuity below 6/60. Comparison of the proportion of visual acuity better than 6/60 on the Snellen scale at initial or follow up evaluation did not show significant differences between dialysis and control group. However, more patients with endogenous endophthalmitis in control group achieved improvement of visual acuity than the dialysis group (*p* = 0.012). The odds ratio (OR) of no improvement in visual acuity of the dialysis group compared to the control is 9.8 (95% confidence interval: 1.1–83.9, *p* = 0.037). The outcome of exogenous endophthalmitis was better than endogenous in the control group (*p* = 0.015).

**Table 5:** Visual acuity before and after treatment for chronic dialysis patients versus patients with normal renal function

	Dialysis				Control			
	Endogenous (*N* = 25)		Exogenous(*N* = 7)		Endogenous (*N* = 31)		Exogenous (*N* = 26)	
Initial visual acuity								
Below 6/60	15	60.0%	4	57.1%	25	80.1%	22	84.6%
Better or equal to 6/60	3	12.0%	1	14.3%	6	19.9%	4	15.4%
Unable to assess/data missing	7	28.0%	2	28.6%	0	0%	0	0%
Follow up visual acuity								
Below 6/60	19	76.0%	3	42.8%	24	77.4%	12	46.1%
Better or equal to 6/60	2	8.0%	2	28.6%	7	22.6%	14	53.9%
Unable to assess/data missing	4	16.0%	2	28.6%	0	0%	0	0%
Outcome by visual acuity								
Improvement	1	4.0%	1	14.3%	9	29.0%^#^	15	57.7%^*^
Stable	11	44.0%	3	42.8%	9	29.0%	7	26.9%
Deterioration	4	16.0%	1	14.3%	5	16.1%	2	7.7%
Enucleation	4	16.0%	0	0%	6	19.4%	2	7.7%
Unable to assess/data missing	5	20.0%	2	28.6%	2	6.5%	0	0%

^#^ *p* = 0.012, Endogenous: Dialysis v.s. Control

^*^ *p* = 0.015, Control: Exogenous v.s. Endogenous

The blood and vitreous cultures were listed in Table 6. In the endogenous group, only three patients had positive blood and vitreous culture with same microorganisms. *Staphylococcus* species, *Klebsiella pneumoniae* and *Pseudomonas* species are among the most common isolated pathogens of endogenous endophthalmitis in the dialysis group. In control group, *Klebsiella pneumoniae* was the most common pathogen identified in those with endogenous endophthalmitis, and most of these patients suffered *Klebsiella pneumoniae* bacteremia from various focus including hepatic, renal and retroperitoneal abscesses; while Coagulase-negative *Staphylococcus* and *Enterococcus faecalis* were the most common pathogen causing exogenous endophthalmitis in the control group.

**Table 6:** Microbiological features of endophthalmitis in chronic dialysis patients

	Dialysis				Control			
	Endogenous (*N* = 25)			Exogenous (*N* = 7)	Endogenous (*N* = 31)			Exogenous (*N* = 26)
	Blood only	Vitreous only	Blood + Vitreous	Vitreous	Blood only	Vitreous only	Blood + Vitreous	Vitreous
Gram positive bacteria								
*ORSA*	4	1		1	1			
*OSSA*	1				1	1		
*CoNS*		2	1			1		6
*Staphylococcus epidermidis*								1
*Staphylococcous homonis*								1
*Streptococcus pneumoniae*						2		
Group B *Streoptococcus*					2			
Streptococcus oralis								1
*Enterococcus faecalis*		1		1	1			3
*Aerococcus*						1		
*Bacillus cerues*	1							
Gram negative bacteria								
*Klebsiella pneumoniae*		3				3	4	
*Pseudomonas aeruginosa*		1	2					2
*Pseudomonas fluorescens*		1						
*Morganella morganii*								1
*Stenotrophomonas maltophilia*				1				2
*NFGNB*				1				
*Myroides odoratus*								1
*Ochrobacterium sp.*	1							
Fungi								
*Acremonium*		1						
*Aureobasidium*								1
*Candida parasilosis*						1		1
*Fusarium*								1
*Pseudoallescheria boydii*						1		
*Scopulariosis*								1
*Trichosporon*								1

*ORSA* oxacillin-resistant *Staphylococcus aureus*, *OSSA* oxacillin-susceptible *Staphylococcus aureus*, *CoNS* Coagulase-negative *Staphylococcus*, *NFGNB* non-fermenting gram negative bacilli

## Discussion

Endophthalmitis is a serious condition that may leads to blindness, for which prompt management may be associated with the vision outcome [1, 2]. It can be generally divided into endogenous or exogenous. The exogenous endophthalmitis is more common, and can be resulted from surgery, trauma or extension of ocular surface infection. The endogenous endophthalmitis is rare, accounting for only 5–10% of the endophthalmitis population, and is mostly caused by secondary inoculation of microorganisms from infective foci such as wound, endocarditis, liver abscess, and urinary tract infection, etc. [1, 2, 7]. In our case series, the prevalence of endogenous endophthalmitis is higher than exogenous endophthalmitis in chronic dialysis patients.

Chronic dialysis patients have defects in both innate and adaptive immunity, thus predispose them to infectious disease [12, 13]. However, only a few case reports described endogenous endophthalmitis of dialysis patients in the literature. These case reports highlight that vascular access should be considered as a possible and unique infection source in dialysis population [15–18]. In a retrospective study in Denmark, the incidence of first episode of bloodstream infection in dialysis patients is higher than that in population control [19]. This group also demonstrated a very high incidence of *Staphylococcal* bacteremia in dialysis patient [20]. Studies from Canada also found that dialysis is a strong risk factors of both *Pseudomonal* and anaerobic bacteremia [21, 22]. Because of the higher incidence of bloodstream infection, the dissemination of bacteria into various organs becomes an important issue. In studies focusing on general population with endophthalmitis, the most common pathogens are *Staphylococcus aureus* [3, 5, 6, 8, 23], *Klebsiella pneumonia* [3, 6, 7], and *Candida* species [5–7]. In our case series, the main bacteria isolates from dialysis population include *Staphylococcus* species*, Klebsiella pneumonia, and Pseudomonas aeruginosa*; while in the control group, the most common pathogens include *Klebsiella pneumonia*, *Staphylococcus* species, and *Enterococcus faecalis*. The most prominent difference is the presence of *Pseudomonas aeruginosa* and this implies the necessity of anti-pseudomonal coverage in empiric treatment for endogenous endophthalmitis in chronic dialysis patients.

Chronic dialysis patients are also frequently subjected to bacteremia because of vascular access infection [24]. In our study, 4 of the 25 patients diagnosed with endogenous endophthalmitis have documented vascular access infection. All of them have arterio-venous grafts or tunneled cuffed catheter, which is compatible with previous studies indicating higher risk of bloodstream infection when using vascular access other than native arterio-venous fistula [25, 26]. However, because of the lack of concordance in blood and vitreous culture in three of four dialysis patients, we cannot conclude the causal relationship between the vascular access infection and endogenous endophthalmitis, but only address the possibility of unique bacterial source in the dialysis group.

The outcome of endogenous endophthalmitis is poor but with some improvement over the last two to three decades [3, 4]. Compared with more recent endogenous endophthalmitis in general population and our control group, the dialysis patients may have poorer initial visual acuity and less favorable visual outcomes [5, 6, 8]. This may be explained by higher proportion of diabetes mellitus (72%) in our group than in studies of general populations (29 ~ 62%) [3–8, 23]. The higher proportion of diabetes mellitus in out chronic dialysis patients can explain more prevalent diabetic retinopathies before the index episode of endophthalmitis. The initial visual acuity is associated with final visual outcome, as shown in a retrospective study by Nishida et al. [8]. Any delay in diagnosis will possibly lead to worse visual outcome [3, 4, 23].

We found 7 dialysis patient had exogenous endophthalmitis after surgery. Six patients are male and six patients have diabetes mellitus (DM). Male gender and DM have been described as patient-associated pre-operative risk factors for post cataract surgery endophthalmitis [11]. Based on previous studies and meta-analysis, prophylaxis with intracameral injection of cefuroxime reduce the rate of post cataract surgery endophthalmitis [27–29]. Because our patients underwent cataract surgery in other ophthalmologic clinic, we were not aware of the use of prophylaxis antibiotic during the surgery. The visual outcome of exogenous group is better than endogenous group in our patient series (proportion of final visual acuity better than 6/60: 28.6% v.s. 8.0%). Although more patients in the control group achieve visual acuity better than 6/60, the difference is not statistically significant (control: 53.9% v.s. dialysis: 28.6%, *p* = 0.235). By comparing the proportion of final visual acuity better than 6/60, the dialysis group with exogenous endophthalmitis has worse outcome than that in the general population. Choi et al. reviewed the outcome of post-cataract surgery endophthalmitis in Asia and found the final visual acuity better than 20/200 were between 31.6 to 100% [30]. In a nationwide 6-year cohort in Sweden, Friling et al. reported the proportion of final visual outcome better than 20/200 to be 66.6% [31].

## Conclusion

To our knowledge, this is the largest series of endophthalmitis in chronic dialysis patients and provides important information to the nephrologists. Firstly, as compared with general population, the prevalence of endogenous endophthalmitis is higher than exogenous endophthalmitis in chronic dialysis patients. Second, *Staphylococcus* species, *Klebsiella pneumoniae* and *Pseudomonas* species are among the most common isolated pathogens of endogenous endophthalmitis. Also, the vascular access should be closely evaluated because it may be a possible focus of bacterial entry in chronic dialysis patients. Third, the visual outcomes of endophthalmitis in dialysis patient may be worse than general population. Because of the potentially dismal outcome, high alert of eye symptoms in chronic dialysis patients and prompt referral to ophthalmologists are suggested for the salvage of visual acuity.

The limitation of our study includes small case numbers that may lead to inadequate power to detect smaller differences between the variables. The retrospective chart review provide evidences of association, but may not imply causal relationship.

## Abbreviations

AVF: Native arterio-venous fistula
AVG: Arterio-venous graft
CF: Count fingers
CoNS: Coagulase negative *Staphylococcus*
CRP: C-reactive protein
DM: Diabetes mellitus
ESRD: End stage renal disease
HCC: Hepatocellular carcinoma
HD: Hemodialysis
HM: Hand movement
IVI: Intra-vitreous injection
Kp: *Klebsiella pneumoniae*
NFGNB: Glucose non-fermenting Gram negative bacilli
NPL: No perception of light
ORSA: Oxacillin-resistant *Staphylococcus aureus*
OSSA: Oxacillin-susceptible *Staphylococcus aureus*
PAOD: Peripheral arterial occlusive disease
PD: Peritoneal dialysis
PL: Perception of light
PsA: Pseudomonas aeruginosa
SLE: Systemic lupus erythematosus
TCC: Tunneled cuffed catheter
TPPV: Trans pars plana vitrectomy
UTI: Urinary tract infection
